# Between harm reduction, loss and wellness: on the occupational hazards of work

**DOI:** 10.1186/1477-7517-10-5

**Published:** 2013-04-01

**Authors:** Benjamin C Shepard

**Affiliations:** 1 Human Services Department, New York City College of Technology, City University of New York, 300 Jay Street, Brooklyn, NY, 11201, New York

**Keywords:** Suicide, Self injury, Secondary trauma, Vicarious trauma, Wellness, Overdose

## Abstract

Those working in the fields of harm reduction, healthcare, and human services must cope with a range of stresses, including post traumatic stress and vicarious trauma. Pain and loss are just a part of the job. So is dealing with premature death as a result of HIV, hypertension, and even overdose. Faced with a range of challenges, some workers in the field even turn to self-medication. For some, it is about pleasure; for others it is about alleviating suffering. In recent years, several leaders in the AIDS and harm reduction fields have died ahead of their time. Some stopped taking their medications; others overdosed. Rather than weakness or pathology, French sociologist Emile Durkheim saw self-destructive behavior as a byproduct of social disorganization and isolation, as a way of contending with a breakdown of social bonds and alienation. There are any number of reasons why such behavior becomes part of work for those involved with battling the dueling epidemics of Hepatitis C, HIV, and related concerns. Forms of stress related to this work include secondary trauma, compassion fatigue, organizational conflict, burnout, complications of direct services, and lack of funding. Faced with day-to- day struggles over poverty, punitive welfare systems, drug use, the war on drugs, high risk behavior, structural violence, and illness, many in the field are left to wonder how to strive for wellness when taking on so much pain. For some, self-injury and self-medication are ways of responding. Building on ethnographic methods, this reflective analysis considers the stories of those who have suffered, as well as a few of the ways those in the field cope with harm and pain. The work considers the moral questions we face when we see our friends and colleagues suffer. It asks how we as practitioners strive to create a culture of wellness and support in the fields of harm reduction, healthcare, and human services. Through a brief review of losses and literature thereof, the essay considers models of harm reduction practice that emphasize health, pleasure and sustainability for practitioners.

## Background

Every year, I devote a couple of weeks of my undergraduate course on community mental health to the perils of traumatic stress, vicarious trauma and the imperative for harm reduction and human services practitioners to assess their own capacities for health and wellness over the long term. Throughout these discussions, I find myself running through an inventory of supervisors and colleagues who have suffered through hypertension, stress, depression, overdose, and even untimely death while involved in the practice. The eyes of some of my students grow wide. It is not easy to put a finger on why some shuffle off earlier than others or why we inflict wounds on ourselves. But many of us do, sometimes fatally. The list of casualties grows longer with each passing year. Like many, I wonder how to make sense of these losses or prevent them.

Various writers and social theorists have attempted to grapple with patterns of ill health among those in health care and services related fields [1]. In *Beyond the Pleasure Principle*, Freud described self-harming behavior as part of a Thanatos syndrome [2]. Just as many of us have an instinct that leads us toward pleasure (or Eros), the flip side is a ‘death instinct’ which compels us to engage in risky behavior, involves a degree of release. In some cases, this space involves personal freedom; in others it involves a drive to find some peace or relief from pain and suffering [3]. These forces contend with each in countless ways [4-7].

The concept of risk includes countless meanings worth engaging and striving to make sense of. Some see it as a byproduct of byproduct of social disorganization and isolation, a response to limited social integration or solidarity. For many, risk is a way to connect with others engaged in the same activities. For others, it is a means to a sense of fun, a thrill in taking part in activities which may end up hurting us. Sometimes looking at death in the eye makes us feel more alive. Many risk-takers advocate getting the most they possibly can get out of life while turning away from the bland middle of the road. Here, the capacity to elude death or injury through their own preparation, skill, and knowledge provides a feeling of agency, a sense of greater control and mastery. Some of these forms of risk offer powerful social rewards and the possibilities for joy, as well as sanction for activities deemed antisocial. While these types of risk produce similar feelings of personal fulfillment, the former seems to be motivated by a sense of care for other people and ideas, while the latter seems to result from anger, alienation, or disaffection. For many, risks are a challenge to a status quo bent on multiple social controls [3,7-11].

Rather than weakness or pathology, French sociologist Emile Durkheim saw self- destructive behavior as a byproduct of social disorganization and isolation; it was a response to limited social integration or solidarity [8]. In this respect, it is a way of contending with a breakdown of social bonds and alienation. This breakdown takes any number of forms for those in social services. Few of our agencies are immune from the competition of any other workplace [12]. These days, social services must do more with less funding. Thus, “squabbling and backbiting” are just part of the work in many agencies [12]. Some people end up feeling undervalued [13], bitter, isolated [14], overwhelmed with guilt, or unable to reach out for care [15].

In New York City, the majority of people accessing survival services are people of color. Issues of race and immigration status impact practice in countless ways. Participants who search out services experience racism and stigma throughout their lives. Racism impacts interpersonal working relationships, and thus becomes central to the work of harm reduction in a very real ways. This creates still more stress. Most of this stress takes place while those in the field vicariously take on the trauma long experienced by social outsiders such as drug users, undocumented persons, the homeless, people with Hepatitis C and HIV, sex workers, and sexual outlaws. Sometimes we even have to deal with our colleagues engaging in self-destructive behavior while simultaneously helping others cope with their own pain [16].

“Many harm reductionists have made a conscious decision to put some of the most difficult feelings and emotions, those of oppression, marginalization, and political disenfranchisement in our personal backpack, and these are heavy loads to carry,” notes Rafael Torruella, the executive director of Caim Fajardo, a syringe exchange program in Puerto Rico. “And we carry these loads because we are in many senses, a stop-gap for society’s ills” [17].

Many workers develop a profound sense of empathy for with whom they work. Others are drawn to harm reduction work because of its larger mission, as well as associated feelings of social integration and solidarity born of the struggle against larger social injustices [18]. This is a form of services work with feels raw and vital, drawing in those from a wide range of settings, looking for acceptance, care, and a way to fight back against punitive systems. Yet, it is by no means easy work.

Those who work in harm reduction, health care, and human services related fields experience multiple levels of stress, ranging from structural violence to persistent poverty. Some of this stress comes from direct services, including efforts to fund their organizations [19,20]. Accumulative forms of stress include secondary trauma, compassion fatigue, organizational conflict and just plain old burnout [1,15,21-23]. Yet many of the coping techniques used fail to provide adequate relief from the day to day stressors of work [23]. How do we strive for wellness when we are taking on so much pain, particularly when working in an often death saturated field, such as harm reduction? For nearly two decades the harm reduction movement has attempted to provide support for those coping with the stress of the work, yet support has not materialized to match the scale of the need. Certainly there are structural reasons for this. Poverty has only increased nationally, while safety net provisions have been forced to do more with less, placing stress on those on the front lines.

The impacts of these difficulties are far reaching.

“This is the most problematic part for me in thinking about these issues,” notes attorney Corinne Carey, who has been involved in harm reduction for nearly two decades. “These questions come to the fore when people with whom we share extremely close bonds and solidarity die” [18].

Most certainly, harm reduction works. It helps reduce the spread of HIV / AIDS among injection drug users [24]. It helps gives us tools to engage those who otherwise would be lost to care [7,25]. Yet its linkage between drug use, high risk behavior, and illness makes it difficult work, exposing workers to multiple risks including secondary trauma. This is a risk which we sometimes take for granted.

The following considers a few of the ways we cope with harm and pain, as well as the moral questions we face when we see our friends and colleagues suffer or inflict pain on themselves. This paper considers themes of: harm reduction and risk, limits of non-profit models, recommendations for practice, the difficulties of saying goodbye, and ways to build a culture of wellness into harm reduction. The aim of the paper is not to condemn or blame anyone or any organization as much as to reflect upon what happens to those we lose and how can we do better moving forward? Reviewing both the literature as well as a litany of losses, this paper asks what happens when we see our friends and colleagues suffer? How can we support friends and colleagues when the pain of this work starts to consume them? How can we create a culture of wellness, pleasure, care, and support in the fields of harm reduction and human services?

### Methodology

This paper builds on my experience as a long term participant observer, supporter, and advocate of harm reduction. I was inspired to write the paper after a bike accident which was largely self inflicted. Sitting down on the couch for a week I had planned to spend on the road, I thought about the pain I felt, which I had caused. I also read that a friend from harm reduction had died of an overdose. All the pain, hope, and heartbreak from my previous career in harm reduction came rushing back to me. Full disclosure, from 1993 to 2005, I served as a practitioner in various harm reduction settings, including at the syringe exchange program referred to in this report; since then I have worked as an educator and board member writing and teaching on materials related to harm reduction, loss, and human services practice. Many of these experiences are incorporated into this ethnographic report. Through participant observation, this paper report makes use of the researcher’s feelings, thoughts, and reflections as subject of consideration in and of themselves. This form of writing invites readers into the personal and emotional subjectivities of the author [26]. This method is a useful match for a reflective analysis such as this, which touches on emotional impacts of premature loss and the implications of practice. Like all ethnography, this report builds on cross references and comparison of multiple forms of data including personal observation, narrative, and secondary sources, as well as correspondence and short interviews with other service practitioners. In this way, contradictions are reconciled through comparison or triangulation of multiple data sources [27].

### Discussion - harm reduction, risk and Thanatos

Michael Carden saw harm reduction as a means of coping with occupational safety; it was a way to struggle against the dangerous politics of prohibition and criminalization [28]. Its non-judgmental ethos, as well as low-threshold approach to reaching out to those on the margins, provide basic survival supplies, education, counseling, and prevention tools, which helps it achieve maximum impact, argued Carden [28]. This outreach to outsiders gives it a certain moral character, which Carden admired. I knew Michael when he helped run our syringe exchange program at CitiWide Harm Reduction in the early 2000’s, later connecting with him at fundraising parties for the Washington Heights Corner Project, where he served as co-chair of the board. Over the years, we talked about Durkheim, ethnography, drug use, counter culture, politics, ibogaine, and music. He always welcomed me with a kind hello. But plans to hang out socially never really panned out, despite our best efforts. We could not pull off actually meeting up outside work. Carden helped execute the community needs assessment supporting Washington Corner Project’s New York State Syringe Exchange Program waiver application. During his day job, he served as a Project Director at SUNY Downstate Medical Center in Brooklyn and had a joint position at The Center for the Study of Hepatitis C at Weill Cornell Medical College. There he built on 10 years experience designing, implementing and evaluating community-based programs providing medical and supportive services to people who use drugs. He was an advocate of providing health, hepatitis C medical care, and antiviral treatment to active drug users. He was also an active drug user himself.

On Monday April 9th, 2012 Michael Carden was found dead at the age of forty [29,30]. Hearing the news, I was immediately shocked but also painfully aware that harm reduction has its limits. I was angry, frustrated, and saddened when I heard what happened. And I certainly was not the only person who knew him who felt this way [16,31].

“He was an important friend who I trusted a lot,” wrote his colleague Emily Winkelstein shortly after his death.

“[I]ts hard to write this because it just really sucks that he died. It sucks that he was struggling – with his demons and his drug use. And it sucks that Michael died of an overdose – and knowing that no matter how hard we work, and no matter how careful we are, sometimes there are still accidents. I miss you so much Michael… thanks for being my friend,” [16].

Sitting with the anger, with the hurt, and the confusion was part of what inspired me to explore the way that the harm reduction movement copes with these losses.

This, of course, was not the first time a leader in the movement had shuffled off before his or her time. Brian Weil, who started CitiWide Harm Reduction, where Michael and I both worked, died of an overdose in 1996 [32,33]. Psychologist John Watters, who wrote a 1994 report on the ways syringe exchange reduces the spread of HIV/AIDS, died of an overdose in 1995 [34,35]. Harm reduction innovator Rod Sorge died at the age of thirty in 1999 [36]. Sorge was an active part of the ACT UP syringe exchange group which helped lay the groundwork for the legalization of the practice in New York, successfully leading a field committee through use of direct action to promote the intervention as a ‘medical necessity’. Still, for Sorge, heroin use was part of pain management in a lonely world [6]. Sorge, Weil, and Waters are not exceptions. Leaders in the field including Angela Daigle, Jon Paul Hammond, Matty Love, Pete Morse, Sheila O’Shea, and Nelly Velasco all suffered similar fates, shuffling off before their time. Others, such as AIDS activists Dr. Ramon Torres and Spencer Cox struggled with drug use. Cox eventually stopped taking his HIV medications and Torres’ career was severely impacted [37,38]. Many of these departures are akin to the loss of family members for those in the AIDS activist, harm reduction communities where such losses are loaded with social and moral stigmas, as well as intense feelings of frustration, guilt, anger, shame, helplessness, and questions about whether those in the community could have done more [39].

Take the loss of Nelly Velasco, a 19-year-old harm reductionist who overdosed and died on October 9th, 1996. Shortly before her death, Valasco presented at the first Harm Reduction Conference in Oakland and later published her paper “Nelly Valasco is in Control” (as did Rod Sorge, and others) in *Harm Reduction Communication* in the Spring of 1997 [40]. In it, the author confessed to struggling with social condemnation over her drug use, at home and even at work. She noted she needed support and often got it. Yet she fought to cope. The article was followed by an epitaph, noting that Valasco had overdosed just after the Harm Reduction conference in Oakland [41]. “…And man, did we have questions then!” recalled Corinne Carey [18]. Subsequent issues of *Harm Reduction Communication* carry article after article about harm reduction and occupational health. The anonymous author of, “On the Death of Nelly Valasco,” confesses to only finding out about the loss after reading the previous edition of *Harm Reduction Communication*; yet the anonymous author finds herself gripped by questions about the loss [42]. “Was it a suicide?”, the author's friends ask. Regardless, “we all need to start to understand what fucking role harm reduction plays in our lives,” the anonymous author continues. “I don’t want to see another one of my friends or colleagues in this movement die” [42]. The same issue of *Harm Reduction Communication* included an article by Lisa Moore on self-care and the movement [13]. In it, Moore mused about the direction for harm reduction: was it going to be a bureaucracy or a health movement in support of radical public health? And how could the movement create a new kind of organizational culture truly supports self care among practitioners [13]. Another writer in the issue begged the question: “How Many More Deaths before We Come Together?” [14]. It implored those in the movement to try to listen, care, and respect each other a little bit more. Letter after letter to the editor poured in.

Firstly, I wanted to respond to the article written by “Anonymous” regarding the untimely death of Nelly Velasco. I appreciated Anonymous’ visceral and honest response to the news of our loss of her. The message of caution and self-care cannot be overemphasized--and should be reiterated so much that we, in the harm reduction movements and non-users, hopefully, internalize it. Some years ago when I was using, it took me two near-fatal overdoses to get the points Anonymous is making [43].

After all, people use drugs for multiple reasons. And drug use involves multiple meanings worth understanding, Michael Carden used to remind me. Our job was to make sense of these motivations, desires, and needs.

Over the next few years, many would reflect on the meanings of their own drug use and the ways they navigated it with work in the issues of *Harm Reduction Communication*, a peer based journal open everyone. Take Johanna Castilla.

“Like so many women, I still struggle with issues of my physical self,” noted Castilla [4]. “In the past I would sacrifice my health just to be thin.” Heroin was a way to cope, even though Castilla knew it had its downsides. “It helped me detach from a painful world of suffering and a life void of true importance and meaning, a product of our western anomie” [5]. Durkeim [8] argued this “anomie” was part of the compulsion to harm one’s self. These feelings often accompany those in the field through their efforts to prevent overdoses and cope with HIV and structural violence, just as premature mortality follows drug users who participate in harm reduction programs themselves [44].

Over the years, the harm reduction movement has effectively engaged in a range of preventive practices ranging from HIV to overdose, democratizing the use of the opioid antagonist naloxone [24]. Routinely used in hospitals and by paramedics to revive those overdosing on opioids, since 1996 harm reduction organizations have been training users, peers, and programs to respond to overdoses, as well as to use naloxone. In the last 15 years, 188 local, take-home naloxone programs reported 10,171 drug overdose reversals. Over the same period, drug overdose deaths increased three-fold. “Thousands of fatal overdoses occur every year,” notes Eliza Wheeler, author of the study, *Community-Based Opioid Overdose Prevention Programs Providing Naloxone — United States, 2010*[45]. “[B]ut … we can reduce overdose deaths by giving members of the community the right information, training and tools,” [45].

Risks are many for both harm reduction staff members and drug users [4]. One of the core challenges of this peer-based model is the ways it requires workers to learn boundaries related to work. Boundaries in harm reduction and AIDS activist communities are often porous, with former users providing trainings, services, and research for programs, where their friends and colleagues are sometimes clients. When practitioners are caring for various clients and struggling to care for themselves, it is more challenging to face the struggle of a friend or a co-worker who becomes a third, fourth or tertiary concern group. Maintaining boundaries is a messy part of this work and a challenge for everyone involved, including drug users, therapists, and outreach workers alike. To the extent that drug abuse is more relevant in harm reduction because the field is more likely to hire peers or others with a history of drug use or drug addiction, it is the collective community’s responsibility to keep individuals healthy and well. And this is not simple.

“A central problem with drug use/users and overdose is the unpredictability of illicit substances,” notes Brooklyn College Sociologist Naomi Braine [46]. There are drug interactions and drug/alcohol interactions; there are a varying degrees of the purity of drugs. “No one knows exactly what they are getting, and that plays a role in unexpected mortality among regular users. In thinking about harm reduction workers and OD, one of the interesting questions becomes how hard it is to apply what we know to our own practices?” [46]. There are personal challenges related to drug use. And there is toying with suicide; there’s being careless; there’s feeling guilty about using alone because there’s no one there to administer naloxone. And there is the state response. While harm reductionists have put a great deal of work into building a movement built on the experience of users, drug policies aimed to curtail it tend to foster unpredictability as well as unintended consequences Measures designed by the state to make us safer tend to have the opposite effect. This paradox highlights one of the core contradictions of a neoliberal model of health in the midst of the drug war [4].

“In my work with homeless youth, what I see very often, is that economically impoverished youth, overwhelmingly youth of color, are navigating the horrors of a decimated and extremely punitive set of welfare systems,” explains Craig Hughes, a social worker with a harm reduction program for homeless youth. “Survival behaviors result because people need to survive and don’t have access to what they need otherwise. Traumas resulting from interpersonal experiences are a major factor in people’s lives, but so are traumas resulting from systemic inequities and oppressions. Much of the survival behavior people engage in is an outcome of dealing with a set of service systems, re-crafted in neoliberal fashion, which provide extremely little – often times nothing – while leaning heavily on discipline and diversion. People are actively pushed from accessing help. Much of the harm reduction work I do as a service provider is in reaction to the harm caused by those systems. The burnout in harm reduction agencies comes, at least partially, because there is a safety net that is definitively more about discipline and diversion than anything else. Harm reductionists rarely publicly focus on the limits of the “safety net,” but it actually seems to me to be utterly decisive to understanding staff burnout – when there’s nothing but push-away and harmful welfare systems, the uphill battle in supporting service-users is *both* the difficulties of personal choice as well as dealing with harmful systems themselves” [47].

Throughout my years at CitiWide Harm Reduction, we constantly juggled with the contradictions within the punitive welfare state, the war on drugs, and high rates of violence within these communities. Members refused drug testing or monitoring of their blood by doctors and treatment facilities, seemingly unwilling to submit their bodies to regulation deemed necessary by the punitive welfare state. A client diagnosed with HIV and kidney failure turned down treatment until his health failed and he passed. A transgender client living in a single-room-only hotel was thrown out of the second floor window to her death on the streets below. Countless clients passed from HIV related complications, as well as Hepatitis C. Another client left her new child at home with her husband, went on a five day drug run only to return to find her husband dead from a heart attack. Her son had starved to death. Others faded away seemingly consumed by the hostility, neglect, and violence of the street. Random violence was constant. Our van driver drove to go meet a friend on election night in 2004, only to be fatally shot in the back on his trip. I remember calling a staff meeting and trying to share the news, not knowing how to convey it appropriately. Grief and multiple losses have long been part of work around HIV and harm reduction. Yet outside our rituals, there were few outlets for those involved to collectively grieve or cope [9]. Witnessing these losses, feelings were often messy and ambivalent among staff, many of whom were former clients. Approaches to self care were inconsistent. Many neglected the need for it. The trend is not uncommon [15].

Throughout it all, Michael coped and counseled, as well as provided survival supplies to those coming into our syringe exchange program. I remember one night he borrowed a copy of *Midnight Cowboy* to show at the Friday night syringe exchange-movie night at CitiWide. It took one of our usually jovial members down a melancholy path about his life and world. Michael was there to talk him through it. Yet, what of Michael’s own pain? Even then, I knew he struggled to cope and supported his decision to leave the syringe exchange program. As a sporadic user, he said it was good for him to finally move on from CitiWide and its syringe exchange.

In 2004, I gave a presentation on the CitiWide Model at the Harm Reduction Conference in New Orleans. Charles King started the conference by talking about the loss of his partner Keith Cylar of Housing Works, who had passed after years of struggling with HIV the prior Spring [48]. His open tears were refreshing, yet the pain of Cylar’s loss as well as the countless others was something with which many in the field were struggling. Few really knew how to cope except to get back to work. For many of us, this pattern was simply part of the common ethos in social justice struggles: “you just keep going!” But this trajectory is often very harmful.. The notion of “A Luta Continua” -- “the struggle continues” –pervades the field of social justice. But that perspective and feeling can lead to both burnout and marked ineffectiveness in personal and political involvements [47]. It is part of the reality of this work, a part of the lived reality of the practice.

At my talk at the conference, many members of the audience took part in a long question and answer session. Pete Morse, a needle exchange and overdose prevention educator, was particularly engaging. I had known Morse for years, encountering him in community garden, global justice, and harm reduction circles in New York for nearly a decade. Protests or street parties, for a while there, it seemed like he was everywhere in New York. I could recognize his distinct beard from across a room. Yet, I had never known he was also completing his graduate degree in history. He had never told me about this part of his life. His questions were right on, leaving me thinking, and wondering and reimagining what I would say the next time I gave that talk. In 2007, he too died of an overdose [49].

“Worker abuse is a huge problem,” noted John Zibbell in an article about Morse [49]. The controversial article acknowledged that harm reduction programs produce significant results, reducing overdose and rates of HIV among injection drug users. “Yet needle-exchange programs can exact a toll on those who operate them,” it continued. The article also highlighted some of the limitations that accompany the often underfunded movement. “Staffers typically earn little or no money for working on bleak urban front lines with traumatized users.” Many of these workers were clients at some point. Once employed, they bring their life experience to peer education in the agencies they once attended. Many are run on a shoe string budget, with little room for training or support. In addition, “[t]hose dealing with other factors -- depression, history of drug use or personal stresses -- may find it particularly hard to cope. Drug abuse is “an occupational hazard,” says Alex Kral, a San Francisco epidemiologist” [49]. The emotional strains of work with AIDS, harm reduction, and health care are well documented [1,9,14,15,22]. Yet approaches to handling the ongoing stressors are less forthcoming, so people embrace the stiff upper lip approach and try to push forward. Harm Reduction has always been hard, but it is also life-affirming. Many of us find our own forms of guerilla theology, as well as a sense of camaraderie with those on the front lines. After Carden’s death, Daniel Raymond [29], the policy director for Harm Reduction Coalition, said this: “At its heart, the harm reduction movement is a close knit family of dreamers, radicals, and outsiders, tempering anger with hope, fighting stigma and marginalization with love.” Yet, there are limitations to this social solidarity [20,22]. Many feel, isolated, and unsupported in this work. Some days workers feel a great sense of comfort from the support they receive from each other; other workers feel marginalized, ostracized, or alone.

One of my best friends in the field was Keith Cylar, a social worker and co-founder of Housing Works, who used to hang out and chat with me on long nights out [25]. Such forms of friendship offered the sort of solidarity that made the work feel worthwhile. Over the years, one of the things we talked about was the cumulative losses related to working and living with HIV. One of my fondest memories of hanging out with Cylar was after hearing a talk given by Eric Rofes in 2003. We talked about surviving while remembering those we had lost. Rofes, a veteran of HIV/AIDS and gay liberation organizing, highlighted the importance of creating a culture of wellness and health among gay men’s communities. Rofes’ inspiration for this writing was the near nervous breakdown he experienced while running Shanti Project, a caregiver program for people coping with illness such as cancer and HIV [9]. I had gotten to know Eric during those days. Shanti was my first job in the HIV health field. I witnessed firsthand the ways people in the community tore at each other with anger over their losses. It was a pattern common among AIDS service organizations [14,22].

During those days before protease inhibitors, it was profoundly difficult work. It still is. In the early 1990’s, clients died on a weekly, even daily basis at the housing program where I worked. Some days, I would arrive and find out a client had died at the beginning of my shift and not know what to do with myself for the next eight hours, except wonder what had happened or what was to become of them. I had had almost no training to do this work. To make sense of the losses, sometimes I just walked late into the night after my four to midnight shift, wondering what life was all about. In an effort to empathize or cope, sometimes I put myself at risk, as many of my clients had. I was glad to have a network of support, people to talk with and air out what was going on. Eventually, I found a therapist equipped to help me talk about what I was feeling, someone who I could speak with without fearing being judged. Talking about these ideas, the compulsion to take part in risky behaviors waned.

Certainly, my experiences were not isolated. Some have come to suggest such self-destructive behavior is a result of vicarious or secondary trauma related to the work [21,50,51]. There is a long history of those coping with HIV by doing similar things. Queer theorists Michael Warner and Douglas Crimp, as well as HIV leaders such as Charles King of Housing Works, have pondered why it is that people who know better still put themselves at risk [52,53]. Part of the appeal of harm reduction is it offers a less judgmental approach to complicated questions about human sexuality, desire and risk-taking. The harm reduction approach suggests we create spaces for people to talk about these desires, allowing the unconscious desire to find expression, and develop capacity for protection. After all, those ideas which go unexplored are often acted upon [54]. This is why harm reduction emphasizes honest, open, and frank dialogue.

This authentic approach to risk and self determination is one of the most compelling aspects of the field; it is part of the appeal of harm reduction. Rather than condemn, the field seeks to understand. “The underground world –ruled by its own laws- was a very appealing part of drugs for me,” mused Johanna Castilla [5]. For many, this is a space for agency, a space to navigate outside of models of social control, work, protestant morality, and aspects of the normal order of things [4,55]. “I encountered different kinds of people, different ways of relating to one another that seemed spontaneous, mysterious, risky, and unfamiliar, fascinating and seductive” [5]. For many, the tightrope walk between self expression and risk - is a fertile space. To court pain, loss, or even death itself, this is a place for desire and agency. Sometimes we feel most alive when we are looking down at an abyss. The appeal of such experience is hard to contain [3]. But so is the loss when we stumble. As the AIDS and harm reduction years continued, many would die, leaving workers, activists and those remaining left to pick up the pieces and wonder why they had survived while friends and colleagues had not. My first supervisor in New York died in the fall of 2005, years after losing his lover to HIV. I always thought he died of a broken heart. Many people have. Reactions to these feelings of loss take any number of forms, including panic, self blame, anxiety, fear, guilt, numbness, longing, helplessness, forgetfulness, and slowed thinking. These feelings change people [56]. “Grief will make a new person of you,” noted Stephanie Erickson, “if it doesn’t kill you in the making” [56]. Yet we still do not know enough about what this does to those who work with loss every day.

The last time I presented at the National Harm Reduction Conference was in the fall of 2010 in Austin, Tx. I was presenting on the topic of pleasure in harm reduction [57]. While the topic involved Eros, its doppelganger, Thanatos lingered in the air. Shortly before the conference Jon Paul Hammond, one of the founders of Prevention Point Philadelphia, a seminal syringe exchange program, and a long-time board member of the Harm Reduction Coalition, died of an overdose [58]. His death loomed over the conference, a stark reminder of Morse’s death three years prior. It was a precarious all too familiar feeling which rears its head for those in this movement.

People cope with this work in multiple ways, including self-medicating with alcohol and drugs. The question is when is this too much? And when is it time to support those we know who are using with questions about safety? One of my friends used to say, “My harm reduction from heroin is abstinence.” Certainly, harm reduction recoils at the idea of the Carrie Nation, who destroyed barrels of beer during the Temperance days, or Nancy Reagan types, who beseeched everyone to ‘just say no’ while their desires are suppressed. Harm reductionists recognize the danger in such forms of prohibition. Still, we have to ask ourselves how we talk with our colleagues as they struggle to balance their need for safety with the imperative of expression, including risks or harms related to drug use, grief, general exhaustion, or expressions of trauma related to this work.

Sadly, such choices have never been easy. Brooklyn college sociologist Naomi Braine [46] argues that, “Within harm reduction as a social movement, there has been a long history around having trouble finding useful or constructive ways to address each other’s drug use.” Even in harm reduction, it is rarely easy to discuss drug use or personal problems with coworkers or friends [59]. This difficulty is hardly exclusive to harm reduction. Friends and colleagues alike confessed they were unable to engage musician Amy Winehouse about her drug addiction before her death in 2011 at the age of 27 [60].

When people die, we are left wondering if we could have done more. Yet, everyone deals with grief in their own way. I remember walking downstairs one day and seeing that one of our case manager’s had made a shrine at his work station in honor of those on his caseload who had died that year. It was an in intricate collage of photos, stories, poems, and memorabilia. I said he could go home early that day and offered a few other forms of support. But none of it seemed like enough. Perhaps it is the human condition to wonder what could have been. Towards the end of Michael Carden’s funeral, one observer noted, “we like to say that everything happens for a reason, but actually we just have a need to ascribe a reason to everything that happens.” When our friends die prematurely, we are left to wonder if there was a way to stop the next friend from suffering a similar fate. “Part of what makes finding out about these OD deaths amongst our friends painful is that sometimes it’s hard to figure out what exactly it was… .” notes Corinne Carey. “We wait for the autopsies, we pour over the results, we debate and yell and rage at each other to interpret what happened… . .it’s rarely clear” [18].

### Wellness, choice, and agency

“Every death opens all the old graves,” they used to tell us in early HIV training in the 1990s. They told us this to make us realize that going forward, with each loss we faced, feelings were going to flow from any number of memories. That is exactly what happened when we lost Michael. Another observer at his funeral reflected on how present those old losses become when we lose someone else, even if years separate the losses. Time blurs between experiences with AIDS, overdose, and other premature, untimely departures. Each are similar and unique. While it may seem peculiar to put them side by side, the experience of other kinds of early and seemingly preventable death, this is also true with homicide, suicide, and crazy accidents that just shouldn’t happen, all tend to blend together. Yet coping with them is part of this work. The feelings around these losses becomes part of our inner life and memory, just below the surface of our daily life, ready to bubble upward with the touch of another loss.

Every time I teach my community mental health class, I review these ideas and experiences, emphasizing that we all have the opportunity to take care of ourselves, if we want to and we commit to that. Everyone who enters this field faces a choice about building a career and a life that supports wellness in all its dimensions. After losing Keith Cylar, his partner with Housing Works, Charles King noted that we all have choices when faced with loss. We can either let the despair envelop us or we can choose joy [7]. The same choice can be made with health and wellness. Embracing wellness involves a conscious, deliberate, active engagement with a more satisfying approach to living. Dimensions include physical, environmental, social, intellectual, occupational, spiritual, mental, emotional, and even financial elements of our lives [61]. Attending to each, we set a clear boundary between professional and personal needs. While few of us ever achieve complete balance, the conscious decision to put together the components of happiness constitutes an important step. Finding happiness can be a Sisyphean task [62], but it is worth striving for every day, even if all we are doing is rolling the rock up the hill until it comes rolling back down. Happiness is an elusive notion. It tends to be more of a process than an outcome. And like any process, it takes constant effort.

On April 1, 2012, I opened the *New York Times* to find an article about a Manhattan psychotherapist named Bob Bergeron with an active practice with gay men, coping with HIV, issues of safety, non-monogamy, and harm reduction. His book, “The Right Side of Forty: The Complete Guide to Happiness for Gay Men at Midlife and Beyond” was in the galley stages. Some time around New Years [the beginning of 2012], Bob committed suicide [63]. A psychoanalyst with his career ahead of him, Bergeron had what looked like a promising career. Yet he was consumed with a suicidal impulse that seems to be grasping an increasing number of people from multiple walks of life [64,65]. That same week, a friend recalled a conversation with someone who worked for the Metropolitan Transportation Authority subway repair group. He noted that since the economic crisis, the number of suicides and attempted suicides had skyrocketed, although few are reported because the authority does not want to make these numbers public.

A few weeks later, I heard about Michael’s untimely death. While it is easy to wonder what is it about harm reduction that drives those doing this work to untimely, premature death, these stories indicate that the struggle to live through these crises is one of the most common challenges for all people. Still, thinking about Velasco, Rofes, Sorge, Weil, Carden, Watters, Bergeron, Cylar, Cox, and so on, I was struck by how many of those involved with AIDS work, with harm reduction, shuffle off before their time. After his lover died of the virus, writer, activist and harm reduction theorist Allan Bérubé struggled to find meaning in the loss. “As I write these words, I fill up blank pages in the open book of AIDS,” wrote Bérubé [66]. “But there are more empty pages staring back at me: the most troubling questions that still haunt me: Why did Brian have to die? Why have I survived this long? Why are my friends still dying?” Finishing the essay, Bérubé conceded that he had no more answers than he had begun with. Yet, he relished the spaces he found for grief, as well as the support and comradery he found in communities of support and care in San Francisco. But he also lamented the lost possibilities, communities, and friendships. “These are pieces of my life which don’t always fit together,” mused the author. “But they are helping me to create who I am, to give my life meaning during this epidemic” [66]. Allan Bérubé died at the age of 61 in 2007. When I think of so many who passed before reaching anything close to old age, I’m forced to wonder if a part of their lives already passed when they watched others die as the epidemic raged.

Still, the imperative to take care of the living remains, particularly for those still coping with the challenges of this work. “With every overdose, every rape, every stolen backpack, every beaten up girlfriend, every back-to-town-&-strung-out-again-after-a-year of-doing-so-damn-good kid, the grief continued to build,” noted street outreach worker Rechel McClean, in an essay drafted after leaving her position in a harm reduction outreach program [15]. “In time I felt like I was going to lose my shit from the cumulative heartache.” At her program, McLean bought a book to list the names of the dead from their program. “[W]ith every death it just sank in that the book would eventually fill with the names of kids and friends, loved and lost. I began to wonder, not if anyone else would die, but just who would be next” [15].

Despite these misgivings, many have little to no idea how to leave the field. “The one piece that I feel has always been missing from others’ work on this topic is the phenomenon that many of our friends may have been driven to continue to use because stopping use excludes us from a community to which we feel strongly connected and supported—we keep using long after we want to because we don’t want to lose the community,” notes Corinne Carey. “Also—the concept of ’self care' has always missed the part where someone says: ‘hey, being exposed daily to all this pain, all this horror, all this drug use is not good for me right now (or maybe never again) and after putting in my x years of good work here, I’m going to move on so that I don’t die.’ You know what happens to those people? They disappear with nary a thanks. One of my closest friends Patrick worked at Streetwork on the Lower East Side—the kids loved him and he almost gave his all to the kids. He was a fantastic advocate, a hero to this community—until he realized that he was likely next in line to OD. His drug use had gotten out of control, unhealthy, and unsatisfying. So he moved on. He lives elsewhere and works in another field. But he’s alive. When people die, we say: oh, he was a hero of the harm reduction movement! I never met anyone better than him! He was a tireless advocate! (this is my least favorite one because, hey, guess what, he was tired. Are you kidding me?). Scholarships are established in his name, portraits hang on the walls, buttons are made. When someone like Patrick walks away, people forget he ever existed. There’s a culture that celebrates martyrdom that I think feeds into the whole phenomenon, but no one talks about it” [18].

### Beyond the iron cage and recommendations for practice

Up to now this paper has considered the ways this work impacts those involved. In terms of coping with the field’s inherent challenges, three areas of harm reduction practice are worth considerring:1) workplace support and supervision, 2) challenges related to the non-profit industrial complex, and 3) the culture and organizational practices harm reduction.

While wellness, health, and freedom are part of the goals of our work outcomes, they must also be part of the process. That requires building a culture of health and wellness into our organizations as well as training. Yet, this is difficult because programs are often underfunded as they cope with endemic poverty. Still, those managing programs could help build assessments of staff health and wellness into their work. This means making time and committing to staff health, in addition to meeting contract performance goals. Yet, many of us are so caught in the race for funds or to meet deliverables [12,20] that we feel restrained by program requirements. In other words, many of these managers are caught within the iron cage of despair described by Max Weber. Here, we are rewarded for hard work above all else [55]. But there are costs to such an approach. The work environments of many community-based harm reduction organizations can be physically and psychically unhealthy, often rife with internal conflict [1,12,22]. Attending to workplace support or clinical supervision is a useful component of building a culture of wellness in the field. Yet, many recoil at the idea of clinical support. So, other outlets such as support groups and informal networks become vital. While harm reductionists have long worked to build less oppressive work environments, much of the process begins with caring for those doing the work.

The Soros Foundation report *Harm Reduction at Work: A Guide for Organizations Employing People Who Use Drugs* serves as field guide with useful tools for practitioners and workplaces to reference [67]. Among the many recommendations, it suggests that those building or running harm reduction organizations, “[a]ddress potential factors that may cause employees or peers to engage in more dangerous types of drug use as a result of interactions with high-risk service users.” Ways organizations can do this include group sessions and individual supervision. “Supervisors should conduct regular check-ins with employees to assess whether these factors are coming into play and to offer any appropriate support the employee may need, including time off from work.” After all, harm reduction must be a part of the organization’s process as well as practice. “Make all staff aware that the organization is committed to a harm reduction philosophy—for employees as well as service users,” the field guide recommends. “If drug use becomes problematic for an employee, he should feel comfortable seeking help from the organization.” Encourage drug users to join groups such as user unions, support groups, etc. Such groups help break down debilitating isolation for users and their friends. In terms of “drug use and triggering problems,” the guide proposes that harm reduction organizations make every effort “to foster respectful working relationships between employees who use drugs and those who do not.” This means, both “creating support groups for drug-using employees,” as well as offering “support to assist non-using employees who have trouble coping with coworkers who use drugs” [67].

Many harm reduction programs are run with a peer model, in which former clients with personal experience with HIV or HCV are assigned to help others to cope. Usually people who have suffered a lot of pain are best at this work. But they are also the people who need support in their places of work. Given this, harm reduction programs benefit from integrating internal work-related programs that help workers identify symptoms of trauma. Much of secondary trauma is caused by working with those who have experienced trauma, as many who come into harm reduction agencies have. Discussion of such issues can be a regular part of weekly supervision, involving weekly monitoring of these symptoms. Through such conversations, workers are advised to create a sense of balance in their life. This involves building equilibrium between work, home, personal, and community life. Such balance does not have to be inconsistent with work. It can support it so that employees are able to come to work and thrive. Balance is supported through forms of social solidarity, connection and relationships with both co-workers and people outside of work. Each element helps workers develop coping networks and capabilities for both themselves and those with whom they work. Other supportive interventions include: effective consultation, therapy, and support groups, which include elements of stress reduction, meditation, spiritual renewal, etc. Yet, the process involves more than engagement with work.

To support a culture of wellness, those involved must commit to making personal life interventions that support health, wellness, and the reduction of harm. A few examples of this include exercise, time with family & friends, emotional outlets such as journal writing or therapy, and even a little travel, if possible [68]. Even a little play helps those involved over the long term [6]. Here, workers sustain themselves and find a little pleasure in their day to day practice [57,68]. Organizations in which workers are exposed to trauma are encouraged to reduce the risks to workers through “areas of organizational culture, workload, work environment, education, group support, supervision, and resources for self care” [69]. The point is that wellness involves both a personal and organizational commitment among those involved.

More than anything, building a culture of self care involves those in the movement looking out for each other [13,14], establishing a space for open communication (50 ), and care [13]. For the movement and practice to succeed, those involved need to be able to talk to each other about their own problems. Yet this is not always easy [14,59].

The conflicts between those of different economic classes, or backgrounds, or between cohorts of users, or users and non-users can tear at organizations. At CitiWide, those who used the syringe exchange hung out downstairs, while those who used crack cocaine hung out in the TV room upstairs, their drug use patterns also breaking in terms race.

Rebecca McLean suggests that building a culture of wellness for harm reduction workers can include elements such as:

1. Prioritizing taking care of self.

2. If one is from a privileged background, acknowledge it and move on. After all, “It is important to be an ally to oppressed people without trying to take on their oppression” [15]. 

3. Whether one is using drugs or not, come to grips with your feelings about it.

4. Figure out a way to really talk with colleagues.

5. Keep an eye out for friends and colleagues; bring them to your support group. Help take care of each other [15].

Some of this involves programming and some involves a commitment of workers to themselves.

Yet, the barriers to implementing these recommendations are many. Some express their pain in violent aggressive ways within organizations. In terms of external pressure, the field has been forced to grapple with challenges related to the non-profit industrial complex. Just to survive, many agencies are re-modeled in increasingly corporate/business oriented ways. Accordingly, staff’s personal involvements in drug usage, for example, is less open, reflecting the highly competitive nature of organizational survival. This complicates the work environment in countless ways. With the professionalization of the field, smaller agencies feel less room for autonomy from larger social forces, mergers, takeovers, etc. The first social services agency I worked in, a very queer friendly workplace, was taken over by Catholic Charities, a larger more conservative business. The transaction was not unlike a corporate merger [9,20].

Others see discussion of vicarious trauma as a deflection of issues related to the precarious nature of work in non-profits in general. Very real challenges of cost of living for front line workers are sometimes obfuscated within discussions of wellness. Instead of talking about low wages, managers focus on emotional difficulties, noted one observer who choose to be anonymous. But just as housing access leads to less harmful behaviors, more money and the resulting stability leads to more self-care. So while agencies may emphasize issues such as “vicarious trauma” – and supervisors are trained and must bring it up with their subordinates – there is often very little discussion on the wage-freeze, the refusal by management to give cost of life increases, and the utter lack of job security. Overwhelming work with low wages is a material reality that causes its own serious hardships that can also evince themselves with similar “symptoms” to vicarious trauma. Sometimes pathologization is a form of systematic dominance perpetrated by the people who administer programs to avoid the blunt reality that harm reduction agencies are not immune to exploiting their workers or taking their needs for granted. Much of this pressure stems from the imperative to provide services with a limited pool of funds.

Jamie Favaro founded the Washington Heights Corner Project in 2005 after years of providing underground syringe exchange in Washington Heights. At the 2008 New York City Anarchist Book Fair, she discussed the challenges she faced in navigating funding for her new organization.

A lot of work goes into getting a syringe exchange program started. And I found that through doing that, who I was as an activist and my work really changed. As we were getting the syringe exchange started, everything started to revolve around funding—who’s going to fund us? And I found that the funding streams and kind of the stress on getting funded really changed the work that I was able to do. I found myself increasingly stressed about deliverables, and stressed about my relationships with our funders. And it’s really interesting because I started to really resent funding. Because when you get funded, you’re a lot more accountable to the funder than to the actual community that you’re serving. It’s all about what they want. When you get funding, you’re expected to do certain things with your funding. Funding shapes the work that you do. Not necessarily the work that needs to be done in the community… . I think that’s because we’ve all become so big, and we’ve all become so reliant on our funding and not wanting to piss off our funders [70].

This pressure around funding creates extremely competitive dynamics between organizations. Take Brian Weil, the founder of CitiWide Harm Reduction. Many who knew Brian Weil found him to be a very intelligent, somewhat difficult person. After leaving New York Harm Reduction Educators (NYHRE), Weil started CitiWide in 1994 [71]. The program was framed about an innovative model of outreach to engage hard to reach populations [71,72]. The last time harm reduction activist Donald Grove saw Weil, his head was spinning with stress over managing the data for another program. Weil took the time to help Grove think about and understand the data in a different way. He described the system he had put together at CitiWide, which he presented as unique. The event was both helpful for Grove and self serving for Weil, who took the opportunity to present the material as an extension of his own intelligence [71]. After Weil passed [32,33], those involved with the agency related that Weil’s plan was to take over other harm reduction programs in the area. Weil left a model that was both compelling and competitive [71]. Aggression was a byproduct of the organization Weil helped create; it was also part of a trend in AIDS services [9,22]. This imprint was left on a culture of harm reduction as the movement churned forward. It also became part of the culture and organizational practices of the organizations born of the social movement that created harm reduction.

Many lamented that harm reduction was increasingly a part of the system of non-profits funded by governments, philanthropy, and modern capitalism [12,20]. “Capitalism is a system which encourages people to view each other in competitive ways,” notes another anonymous author describing the occupational culture of harm reduction [12]. The violence of late capitalism finds its way into the very organizations used to challenge it. “Within agencies there is as much jockeying for position, staking out personal turf, gossip and bitter interpersonal strife as might be found in any office in corporate America – perhaps even more so” notes an anonymous worker in a harm reduction agency [12]. After all, the writer confesses, those in harm reduction work in a space where, “suffering, injustice, and misery are a lot of the population we serve (and, for many of us, love) is what is constantly before our eyes. It wreaks havoc with the psyche of even the most dedicated and in control of us to witness this day after day” [12].

While some thrive in the field; many others stumble. Some workers leave of their own volition [15]; others are forced out when they fail to keep up or are unable to handle their drug use or the stress of the ongoing pain. “I called someone I knew from the field and asked for help” noted one former worker at Carden’s funeral, musing about how isolated he felt after he was dismissed from a position in a harm reduction agency. “Maybe I need detox?” he asked one former colleague, who responded. “You’re fucked up.” No one was really there for him, he explained. Durkheim [8] suggests that such a lack of solidarity may be a contributing factor to social isolation; these broken social bonds wear on people, causing stress on minds and bodies. Alienation takes many forms [73]. A worker loses a job and needs support. The worker feels isolated from old colleagues. One colleague confessed that losing that job was the most painful thing that ever happened to her. Her whole sense of self was lost. Turnover is not just part of the work, it is part of life for those in non-profit, AIDS service and harm reduction organizations [20,22]. That does not mean the workers do not still need help. Many do not get it or even know how to ask for it [15]. After all, harm reduction is a business involving contract management, constant deliverables, and competition for dwindling funding contracts. This is something funders and contract managers remind subcontracting agencies all the time. Those who remain in the field are so busy meeting the goals of work that self care becomes yet another task on a daily to-do list [55,71,74].

“As someone working in the field, we have tried so many different ways to incorporate wellness into our work but nothing has stuck,” notes Jamie Favero, the former executive director of Washington Heights Corner Project [71]. “I still eat my lunch in 5 minutes at my desk while typing or looking something up. Maybe this is a New York thing and not necessarily a harm reduction thing (the self care).” Such stressors are ongoing [51].

Still, many harm reduction agencies have worked to create organizations that emphasize wellness as part of organizational culture. This means using staff resources on training and wellness as the Soros Foundation suggests [67]. Yet, the manager’s role is not to be a therapist. Their job is to assess the wellness and health of workers coping with multiples stressors on the job. A manager with a focus on wellness can help create a culture of health among an entire staff, building it into supervision and organizational practices. After all, if staff are not ok, then they cannot be effective as workers. Conversely, healthy working environments help produce positive health outcomes for those in care [1]. Unfortunately, many managers are unwilling or unable to assess the health needs of those doing the work.

Certainly, there are few guarantees when it comes to questions of health or health promotion. Yet, even when the best programs engage the strategies discussed, some people still die, notes Corinne Carey. “Some of the best programs do this,” she cautions. “---but you are engaging a much more personal and much more difficult phenomenon….we can do all those things right, and still experience the death of our loved ones in this field. What happens when this doesn’t work? What happens when you’ve got a super fucking messy situation where you try taking care of someone, but it just. Simply. Doesn’t. Work.?” There are no guarantees with models of health.

### Saying goodbye over and over again

Reviewing these practices and this history, at the bottom of this is the regret for those who are gone. I miss so many of the friends and colleagues lost along the way. I am disturbed to see what happened to them. So many suffer and just cannot get out of their pain. Everyone has different experiences with watching multiple losses. And some agencies, organizations, and movements are better than others at supporting workers through this. The field provides no guarantees from life’s risks, or the difficulties of living with HIV, Hepatitis, and others diseases.

Just before the final reviews of this article came out, news rolled in that AIDS activist Spencer Cox died in December 2012. After hearing about Spencer’s passing, several of us started chatting and dropping notes on Facebook. Eric Sawyer, a founder of ACT UP and Housing Works, suggested a few of us meet at the Stonewall to hang. Long time AIDS activist Spencer Cox was gone after a long battle with pneumonia. Some said he stopped taking his HIV meds. Praying for the dead and fighting like hell for the living are one thing. But sometimes just living is hard. Primo Levy could survive Auschwitz but living with the memories, the reality that he had survived but others had passed, that was another story. He ended up throwing himself down an empty elevator shaft. Cox was one of the veterans of the famous Treatment and Data group from ACT UP who helped push parallel track and an effective research agenda to get drugs into bodies, the right drugs which would help those who had survived the plague and had a fair chance of living. And then he stopped taking his own medications. In the week after Spencer Cox's death, friends, colleagues, and activists around the world pondered his well-lived life and untimely departure. Some lamented the trauma of watching so many close friends lost from a disease that seems to arbitrarily pick and choose who lives and dies [75]. *The New York Times* obit for Cox specifically pointed out:

“Mark Harrington, the executive director of TAG, said Mr. Cox himself struggled with an addiction to methamphetamines. Some months ago, he said, a despairing Mr. Cox had apparently stopped taking his medication.

“He saved the lives of millions, but he couldn’t save his own,” Mr. Harrington said” [37].

Many condemned the AIDS activist’s use of crystal meth. AIDS journalist Laurie Garrett wrote about her anger at both the increased rates of HIV among young gay and bisexual men and Cox’s descent [76]. “I am angry at Spencer for falling down the meth rabbit hole that is claiming the sanity of tens of thousands of gay men in America, making them careless about their own health and callous about the well-being of others” [77]. Garrett concludes:

“So today I am mad at Spencer for falling off all his wagons, gambling with his own life and contracting full blown AIDS. And I’m very angry with those that shout the clarion call of “end of AIDS” in a world that still has no cure for the disease, no vaccine to prevent infection and little more than the hope that millions of infected people around the world will somehow, after they’ve been on these drugs as long as have the Spencers of the gay community, not fall off their respective wagons” [77].

The point is important. Taking a hand full of medications for a lifetime is not an ideal situation. These medications are not a cure. Yet the condemnations of meth use smack of a prohibitionist rhetoric which does not tend to work. The lessons of AIDS activism, queer theory, and HIV prevention activism suggests that prohibitions are dangerous [7]. We have yet to have a frank approach to HIV prevention, and HIV rates are up 20% among gay men. Yet as a generation of HIV prevention activists have pointed out, condemnations do not help us prevent the spread of HIV. They do the opposite. Today, we’re witnessing this first hand. There is a desperate need for people to have a safe space to talk about HIV, drug use, and risk, free of judgment. Instead of attacking drug use or drug users, it is worth asking why they are doing what they are doing. Drug use is part of this world and this life. So is stress. The harm of these has to be acknowledged, managed, and engaged. Cox worked to fight HIV and its lingering effects for decades. And this apparently wore on him, as it does for many involved in AIDS activism and harm reduction work.

That night at the Stonewall, we talked about Occupy, Queerocracy, AIDS activism, kids, and surviving. Cox had pointed out that those who survived AIDS would have to live with memories of those who had fallen, even as they tried to live. “All I do is work,” explained one activist. Another noted that the stress of coping with those years had persisted. Memories fade but the pain remains. So we talked about living and friendship. Eric noted that many people he knows have worked so hard on living and fighting that they can no longer take care of themselves or move beyond the epidemic or the traumatic stress of the pent-up pain.

Spencer Cox himself was well aware of the need for resources for activists to cope with trauma. He founded the Medius Institute to help those activists, such as himself, who knew how to fight, but found living with the memories of their pasts difficult [38]. “These aren’t people who were ticking time bombs to begin with and then skidded off the road. They’re our best and brightest. I can’t tell you how many terrific, smart, hardworking, amazing people I know hit middle age and just lost it,” he explained in a feature in *New York magazine* on the very public self destruction of Dr. Gabriel Torres, another AIDS activist [38].

As the conversation continued at the Stonewall Inn, several talked about the need for old school support for people living with both HIV and the memories of those no longer here, as well as the psychic scars we still carry, just like people who has survived a war. Others recalled the old HIV widower groups for survivors.

“Aren’t those support groups’ weekly meetings still going?” I asked.

“Not really.”

Well, maybe we need to get them back, those old school mutual aid groups, those spaces where people practice care and non alienating humanity with each other.

In the weeks after his death, I spent a great deal of time thinking and writing about the lines between grief, pain, and sanity. Many people and I talked about the ways we do not have enough personal, intimate conversations, or space to talk through, or work through our drug use, risks, pain, or steps out of sanity. Hamlet entertained the idea of insanity in his soliloquies. Some suggest this is how he coped. He explored his crazy by talking it through. Can we really maintain our sanity by exploring insanity, letting its extremes dance off the perimeters of the mind? Maybe this is what crystal is all about? It is what some of us talk through in therapy, in intimate conversations with friends, and in groups. Looking at it, feeling it, and being in that space, thinking about this material provided me with a space to work through some of the confused, frustrated, and despairing feelings that hit when friends shuffle off before their time. We need to be allowed to be crazy, to step off for a second. But what happens if we act instead of contemplating acting? What happens if we stop taking our meds? What if we can’t come back after we’ve floated out to sea? HIV and drug use have always shown us how unforgiving our bodies are.

### A culture of wellness?

The loss of Spencer Cox, of Michael Carden, and so many others offer moments for those involved in this work to take a step back and reconsider the needs of those involved over the long term. To build a culture of wellness, harm reduction may very well have to re imagine its very organizational practices, approaches to supervision, management, and support. This involves grappling with the question as to whether harm reduction is a non-profit, a business, or a social movement. These are certainly not new questions. After the first harm reduction conference, Lisa Moore wondered, “Is this the next social justice movement or is this the next wave of bureaucrats?” [13]. As a business or non-profit, it risks reproducing the isolating social relations that create alienation among workers [12,20,73]. This social strain is where Durkheim [8] located the impulse toward despair. If it functions as a social movement supporting egalitarian social relations and solidarity among bodies, the isolation which propels harm recedes. While it has been forced to cope with institutionalization at the periphery of funding for a program of radical public health, the model’s character as a movement has been neutralized. Yet, certainly many love the movement’s capacity to reject the dangers of social prohibition. But the movement’s capacity to create change is compromised by its close association with the state and other funders. Harm reduction agencies are caught in extremely difficult and acute tension between supporting organizational survival and risky, but necessary, projects of challenging social norms. While this is certainly not a zero-sum proposition, as of today, the movement faces a profound challenges.

Many in the field have already come to recognize that harm reduction is a field built around the defense of pleasure and authenticity, as well as work. This need for pleasure and social solidarity are important parts of the history of this practice [57,68]. They sustain people. Yet, more of is needed. “If I have one piece of advice for young, aspiring activists,” noted Spencer Cox, shortly before he died. “It is to always hold on to the joy, always make it fun. If you lose that, you have lost the whole battle” [75]. This is a point many have come to recognize in reflecting on the history of harm reduction and AIDS activism, especially as the field becomes more of a profession than a movement.

“There is another point I want to make,” Victor Mendolia concluded during a recent panel on AIDS activism. “ACT UP was a very supportive environment.” The group helped him feel OK about getting tested. They also helped him enjoy living life along the way. “It was really, really fun, supporting your whole being… even if people were dying.... you could still dance” [78]. Maybe that is the point, that harm reduction and AIDS activism require a little pleasure, a little joy and solidarity, along the way. We need it to live. Even harm reductionists need to dance. Everyone does [79].

### Conclusions and final thoughts

There are no simple conclusions for the questions this paper has entertained, except that we have to take care of ourselves. In *The Hermeneutics of the Subject*, French philosopher Michel Foucault [80] hints that we are all fools “when we have not yet taken care of ourselves.” The philosopher would die of AIDS himself at the age of 57 in 1984 (probably exposed years before anyone knew what it was). The point of this paper is not to lay blame on a movement or model of intervention, which has engaged so many people that others would chose not to engage. Harm Reduction has never been a panacea, a magic shield for workers, participants, or practitioners. “You cannot serve a dead addict,” practitioners remind those who condemn the model as too permissive. Yet, few models of public health are without their challenges. Many of life’s best laid plans are fraught with unintended consequences [81]. This paper has tried to reflect on questions about what becomes of the people who begin this work. Recall Angela Daigle, who moved to New York in 1997. She went to a harm reduction workshop, volunteered, and found herself in a leadership position in a noted harm reduction program, where she engaged sex workers and drug users. “She went from walking into a meeting to being a policy advocate with an amazing understanding of the issues,” noted a friend and colleague [82]. Two years after that first workshop, she died of an overdose [49]. What happened and what can be done to prevent similar occurrences? What is the impact of the work on those face with so many challenges? And how do we best cope with the overlap between drug use, mental health, and harm reduction? Over and over, the field has had to ask these questions [13]. Self care involves a range of impulses, outlets and expressions. The point of harm reduction is to acknowledge these drives are part of human life. Rejecting the dangerous logic of prohibition, temperance, or repression, harm reduction aims to allow people to acknowledge these desires, engaging in these acts in as safe a way as possible [7]. From methadone maintenance to condoms to syringe distribution programs, the field has largely been successful at protecting regular people from harm. Yet, wellness, health, and notions of care of the self are often paradoxical. Sometimes the very expression one needs to experience is buttressed in danger. Risk has a high degree of allure [11].

Classical Greek mythology is full of references to struggles between pleasure and death. Few of the sailors in Greek myths could make it past the enchanting and deadly song of the Sirens, which lured sailors with seductive songs of Hades. While most knew the ground around them was lined with bones of sailors who had made similar attempts. Even knowing the risk, they did not want to turn away. In all Greek mythology, only Orpheus and Odysseus made it past the Sirens. Orpheus drowned out the Sirens’ song with other music, while Odysseus plugged the ears of his sailors with beeswax, ordering his crew to tie his foot to the mast and tighten the ropes when he asked to be released [62]. The allure of the song was very, very real, Orpheus and Odysseus acknowledged. For many, risk is part of pleasure [4,5,11,45]. This sentiment is something humans have been coping with for a long time. We all want to be part of Lou Reed’s perfect day. For many, drug use and sexuality serve as means of rejection of pain, as well as structures of a normative social order [11]. Pleasure is found in losing one’s self in connection with everything [4,57]. Harm reduction was born with these struggles for safer expressions of these engagements. It is also part of the healthy efforts aimed at the rejection of the dangers of prohibition, in favor of human expression [7]. Over and over it can be seen as a challenge to a status quo bent on multiple social controls [3,7,9-11]. Risk takers and rebels help us point to where we might need to go. “All change in history, all advance, comes from nonconformity,” notes historian A. J. P. Taylor. “If there had been no troublemakers, no dissenters, we should still be living in caves.” The lives of risk takers are worth studying. understanding, respecting and caring for [10]. If the losses recalled in this essay suggest anything, they remind us we are compelled and obliged to support wellness and health whenever and however we can, particularly in the movement’s social relations as well as organizational practices. “We are all our brother’s keepers,” a colleague used to muse during syringe exchange hours. We are compelled to listen when our colleagues and friends are asking for help. Leaving Carden’s funeral a friend mused: “How is it that we reach out to each other? How do we take care of each other? How do we cope with our pain?”

Over the past two decades, the harm reduction movement has come to be recognized as a valid field of public health intervention. This is a field that involves work with populations coping with multiple risks, including HIV, Hepatitis C, domestic violence, homelessness, homophobia, transphobia, sexism, trauma, racism, and structural violence related to neoliberal economic systems, and subsequent increases in poverty. Some have watched the lives of their friends, clients, and colleagues end prematurely. Many have experienced trauma and death first hand [35,45]. Others have tried to cope with work environments plagued with racism, sexism, classism, homophobia, intimidation, disrespect, intense doses of competition (in which their struggle to support others is undermined by harmful atmospheres within the very organizations created to reduce harm) [12,22]. Durkheim [8] suggests there are times when people who feel no social solidarity see few options for living. Those in harm reduction and human services could do well to help those in our midst feel some sense of social connection and community as well as health. We take on a great deal of pain, which in turn, is perhaps the work’s greatest occupational hazard. If harm reduction is to maintain itself as a movement emphasizing health, it would do well to inject an increased dose of wellness and community connection into its own distinct brand of radical public health as well as community organization.

Throughout this reflective analysis, I have attempted to consider the unique culture of harm reduction as an occupation, and its messy connection to a range of social movements. My hope is this piece is the genesis of a larger project looking at the broader narrative of harm reduction work. Still, the limitations of the report are many. It lacks interviews with those across the spectrum of the harm reduction world, or other fields which include large degrees of personal risk. Ideally, it is a contribution to ongoing discussions of radical wellness and pragmatic successful models of self-care. Such interventions are necessary for workers and supporters of harm reduction as well as the larger field of social services, where it is abundantly clear workers need more support. Yet, it is only a small part of a discourse involving a number of interconnected issues that are too complex to be adequately discussed without breaking down further.

Suffice it to say, the Harm Reduction world is rarely one to shy away from the lessons of its losses or ways to learn from them [78]. Already Carden’s mother is using her son’s loss to advocate for more human policies for other drug users. “It’s all about saving a life,” explains Diannee Carden Glenn. “They don’t deserve to die just because somebody zigged when they should have zagged one time um or 5 times doesn’t mean that they deserve to die” [31].

If any conclusion can be obtained from this article it is to remind us we are compelled and obliged to support wellness and health whenever and however we can, particularly in the movement’s social relations as well as organizational practices. Some practitioners bless drug use within the field of harm reduction because we recognize the basic tenants of harm reduction, we allow for variations in Thanatos and Eros; this blessing and openness is what makes working within harm reduction “real” as some practitioners would say. Self-care is not a destination but rather part of a cycle. It is a vital component of taking part in movements that help everyone to feel a little bit more free.

## Consent

All statements provided in this report were included with the consent of the subjects.

## Competing interests

The author declares that he has no competing interests.
